# Combined Treatment with Laser Sintering and Zirconium: A Case Report of Dentinogenesis Imperfecta

**DOI:** 10.1155/2013/745959

**Published:** 2013-03-06

**Authors:** Simel Ayyildiz, Cem Sahin, Özlem Marti Akgün, Feridun Basak

**Affiliations:** ^1^Department of Prosthodontics, Center of Dental Sciences, Gulhane Military Medical Academy, Etlik, 06018 Ankara, Turkey; ^2^School of Dental Technology, Hacettepe University, 06100 Ankara, Turkey; ^3^Department of Pediatric Dentistry, Center of Dental Sciences, Gulhane Military Medical Academy, 06018 Ankara, Turkey

## Abstract

Osteogenesis imperfecta (OI) is a heterogeneous disorder of connective tissue that manifests mainly as skeletal deformity and bone fragility. Dentinogenesis imperfecta (DI) is sometimes an accompanying symptom of OI. The treatment protocol of these patients varies according to the clinical appearance. The case report here describes complete mouth rehabilitation of an 18-year-old male patient with OI and DI using direct metal laser sintering (DMLS) technique of metal-ceramic restorations and zirconium all-ceramic crowns. DMLS is an additive metal fabrication technology that is simpler, more precise, and healthier than conventional manufacturing and can be remarkably cost effective. Moreover, the technique affords highly accurate production of fixed partial dentures with ideal marginal fit and excellent mechanical properties. The patient was treated using a multidisciplinary strategy that focused on controlling caries, protecting teeth from further wear, obtaining an appropriate vertical dimension, and providing soft tissue support to return the facial profile to a normal appearance using new technology in the field of prosthetics.

## 1. Introduction

Osteogenesis imperfecta (OI), also known as “brittle bone”, disease is a genetically determined connective tissue disorder that results from mutations of 2 genes (COL1A1 and COL1A2) responsible for the formation of type 1 collagen [1–3].

Clinical features of OI include growth deficiency, blue sclera, bone fragility, joint hypermobility, dentinogenesis imperfect (DI), and the presence of wormian bones on skull radiographs [3, 4]. Additional radiographic findings are osteopenia, scoliosis, and thin cortices. OI is classified on the basis of clinical and radiologic criteria established by Sillence et al. in 1979 (Table 1) [5–7].

DI is an autosomal dominant form of mesodermal dysplasia that affects both primary and permanent dentition. Shields [8] has identified 3 different types of DI as follows: DI-I: DI associated with OI; DI-II: the same clinical radiographic and histological features as DI-I, but without OI; and DI-III: a rare type of DI found only in the triracial population of Brandywine, MD, USA.

DI associated with OI generally affects primary dentition more severely than permanent dentition. Although the enamel appears structurally normal, it is often dislodged, exposing soft, dysplastic dentin to the oral cavity and provoking rapid, extensive attrition [3, 9]. Histologically, exposed dentin is generally characterized by irregular tubules. Radiographically, teeth affected by DI show cervical constriction, bulbous crowns, short roots, short pulp chambers, and obliterated canals [10]. Adult patients with OI frequently exhibit class III malocclusions, anterior or posterior cross-bite, posterior open-bite, and vertical height loss [1, 3, 11].

Conventional casting is the most frequently used technique for manufacturing Co-Cr alloys for the fixed partial dentures. In recent years, modern computer-aided technologies for manufacturing individual prostheses have been gaining popularity in the field of dental technology [12]. Computer-aided design (CAD) and computer-aided manufacturing (CAM) technologies are used frequently in the dental field to fabricate prostheses ranging from crowns to long-span fixed partial dentures and from removable prostheses to dental implants [13]. The actual fabrication of a Co-Cr product is carried out by computer numerical control (CNC) milling machines or direct metal laser sintering (DMLS) machines [14, 15]. These systems were developed to address a number of disadvantages of the traditional casting method (e.g., increasing cost, manufacturing defaults, and human-aided manufacturing). DMLS is an additive metal fabrication technology that involves the use of a high-power Yb-fiber optic laser [15]. In this technology, metal powder is melted locally with a focused laser beam and fused into a solid part. The parts are built up additively, layer by layer, each of which is 10–30 *μ*m thick [14, 15]. DMLS technology affords highly accurate production of fixed partial dentures with fine marginal adaptation and excellent mechanical properties [13, 16].

This paper presents the multidisciplinary dental treatment of a young patient with DI related to OI. Also, this clinical report explores the use of direct metal laser sintering technique for the fabrication of posterior Co-Cr metal-ceramic fixed partial dentures and zirconium anterior restorations for the treatment of vertical height loss with complete mouth rehabilitation.

## 2. Case Report

An 18-year-old male patient was referred to the Gulhane Military Medical Academy's Department of Pediatric at the Center of Dental Sciences for examination, evaluation, and treatment of defective and hypersensitive teeth. A detailed dental and medical history was obtained. The medical history indicated that the patient had been diagnosed with OI and was being treated orthopedically.

**Figure 1:**
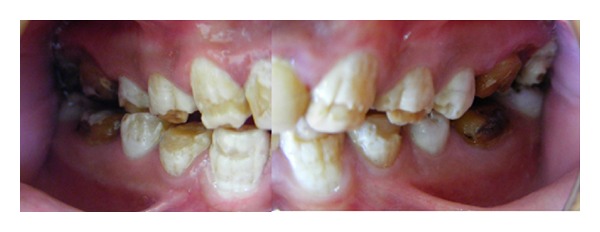
Clinical appearance of extensive enamel deformities chipped during mastication.

**Figure 2:**
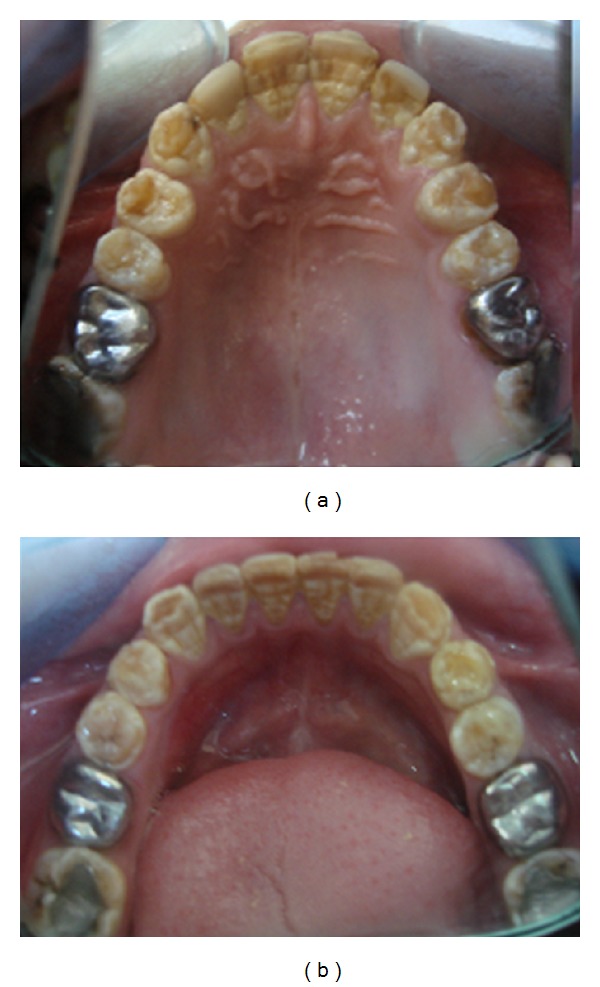
Typical intraoral appearance of dentinogenesis imperfecta. Stainless steel crowns had been applied during mixed dentition to preserve hard tissue.

An extraskeletal clinical examination showed the patient to have moderately short stature, a femoral deformity, and a narrow shoulder breadth. An extraoral examination assessing vertical dimension of occlusion and vertical dimension at rest showed that attrition of the posterior teeth had resulted in an increase in interocclusal rest space (approximately 9 mm). The patient also complained of continuously chipping of teeth in both arches while masticating (Figure 1). Intraoral examination showed that eruption of the permanent teeth was complete and that teeth 16, 26, 36, and 46 had been restored with stainless steel crowns (SSC) (Figure 2). There was extensive destruction of enamel, which was opaque-white in color, whereas dentin was yellowish brown.

**Figure 3:**
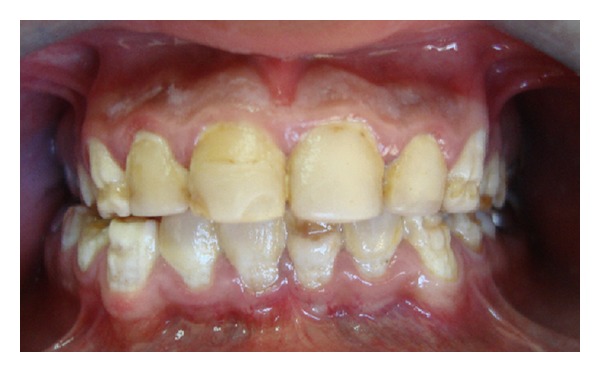
Anterior teeth had been restored with composite laminate veneers for esthetic reasons.

**Figure 4:**
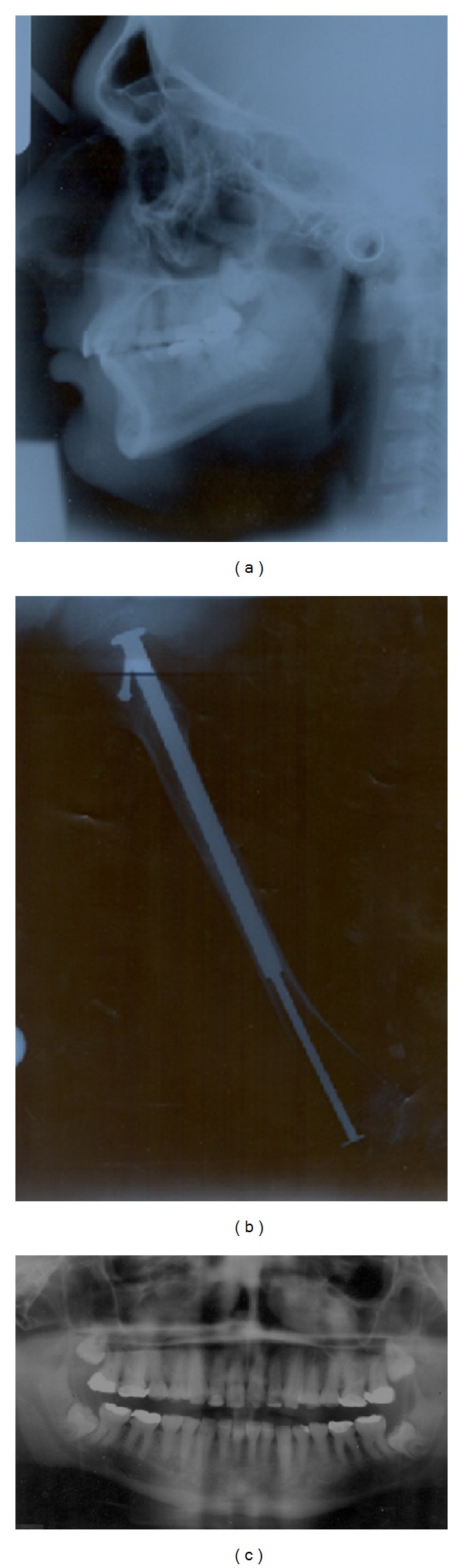
Patient radiographs: Lateral cephalograph (a); plain femoral radiograph (b); and orthopantomograph (OPG) (c).

A dental history indicated that composite laminate veneers had been applied to the upper anterior teeth by a pediatric dentistry clinic for esthetic reasons 3 years previously (Figure 3). The patient complained about general hypersensitivity; therefore, the SSCs were removed, and an OPG was taken. The radiographic examination revealed secondary caries under the occlusal restorations of teeth 16, 26, 36, and 46. There was no evidence of any periapical pathoses. Extraoral posterior-anterior radiographs showed normal skull morphology. A plain radiograph of the lower extremities showed intramedullary rod fixations in both femurs (Figure 4).

Prior to prosthetic consultations, secondary caries were restored with glass-ionomer cement. In consultations with the Department of Prosthodontics, the decision was made to treat all teeth with fixed porcelain prosthetic restorations in order to restore vertical height and esthetics. An occlusal splint was fabricated to reset the interocclusal distance. A TMJ radiograph was obtained with this splint to examine the condyle-articular eminence, and full-time usage of the splint was proposed in order to adjust the dentoalveolar relation to the new vertical dimension.

After 2 months of usage, the splint was divided into two parts anteroposteriorly, and the left and right sides of the splint were used separately to attain the necessary occlusal reduction. A Bis-GMA-based provisional restorative material (C&B Provilink, Ivoclar Vivadent AG, Schaan, Liechtenstein Germany) was used to obtain an interocclusal record of the right side in order to control the occlusal reduction of molar and premolar teeth on the left side.

Master impressions were made with polyvinyl siloxane impression material (Speedex II, Colténe Whaledent Group, Inc, Mahwah, NJ, USA) to fabricate permanent metal-ceramic crown units. Casts were prepared with type III dental stone. After the recording of maxillomandibular relation, the casts were mounted on a semiadjustable articulator (Artex CT, AmannGirrbach, Germany) using face-bow records, and temporary crowns were fabricated in line with the new occlusal height dimensions. Models were scanned with an optic scanner (Activity 102, Smart Optics, Sensortechnik GmbH, Bochum, Germany) and 3D CAD was performed. Direct Metal Laser Sintering (DMLS) (M2, Concept Laser, Hoffmann, Innovation Group, Lichtenfels, Germany) technology was used to fabricate Co-Cr (Remanium Star CL, Dentaurum, Ispringen, Germany) metal frameworks in the 3D Solid Modelling Center at Gulhane Military Medical Academy (3D SMC-GMMA). After metal try-in of the fixed partial dentures, feldspathic porcelain (Vita VM13, Vita Zahnfabrik, Bad Säckingen, Germany) was fired according to the manufacturer's recommendations.

**Figure 5:**
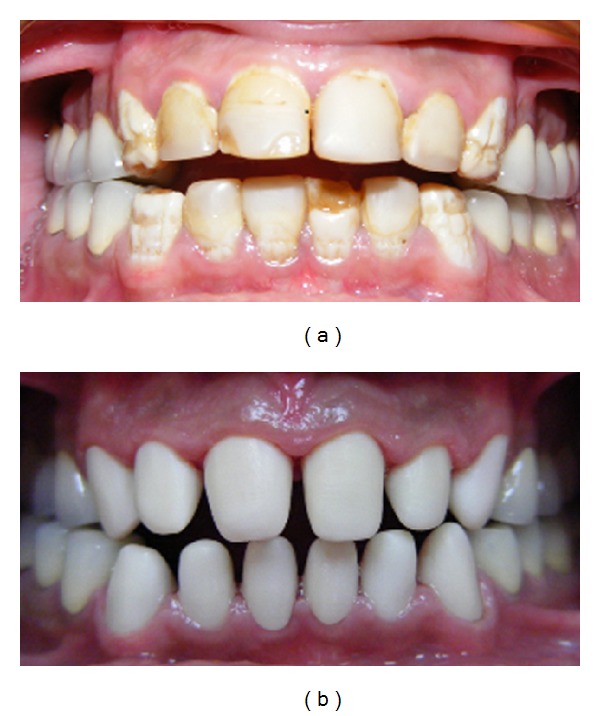
(a) Cemented posterior crowns fabricated from feldspathic porcelain using Laser Sintering technology. Vertical occlusal dimension was established according to the measurements. (b) Zirconium frameworks produced using a 5-axis CNC unit.

Occlusal surfaces of posterior restorations were selectively ground during the porcelain try-in stage to attain mutually protected occlusion at the defined vertical dimension. Restorations were cemented with polycarboxylate cement (Adhesor Carbofine, Spofa Dental, Prague, Czech Republic) (Figure 5).

**Figure 6:**
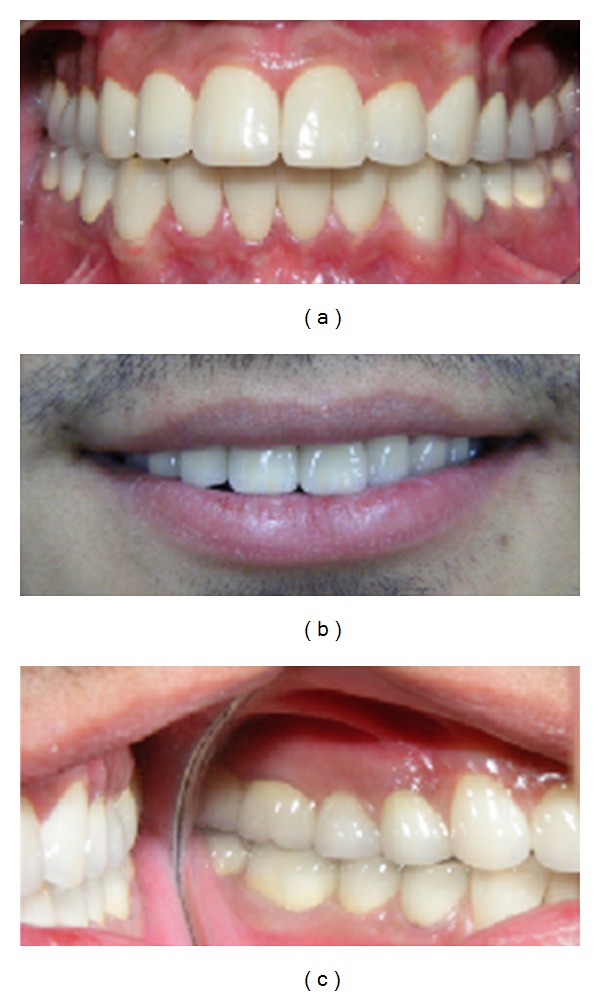
Intraoral appearance of prosthetic restorations.

Anterior teeth were prepared one week after the cementation of molars and premolars. Zirconium porcelain was selected as the anterior restoration material for both esthetics and durability. Impressions were obtained using polyvinyl siloxane (Speedex II, Colténe Whaledent Group, Inc., Mahwah, NJ, USA), and the zirconium restorations were fabricated at SMC-GMMA using a five-axis CNC unit (Figure 5). Restorations were cemented with polycarboxylate cement [17] following try-in (Figure 6).

The patient was recalled at 2-month intervals. Clinical and radiographic examinations revealed no pathoses associated with rehabilitation over a 12-month follow-up period. The patient was satisfied with both the functional and esthetic aspects of the restorations.

## 3. Discussion

OI is a rare disorder that has been reported to be accompanied by DI of varying severity in between 28% and 73% of cases [3, 18, 19]. In such cases, dental, oral and craniofacial variations may be clinically evident [3, 4]. In this case, the patient exhibited signs of OI such as short stature, femoral deformities, and oral manifestations specific to DI.

Skeletal class III malocclusion has been described in many patients with types III and IV OI [3]; however, our patient exhibited an anterior class I occlusal relationship with a unilateral posterior cross-bite. The collapse of occlusal height due to attrition is a typical feature of DI [19] that was present in our case as well. 

Patients with DI may have enamel of abnormal thickness, but frequently is dislodged exposing the softer dentin. Dislodging of enamel may be attributed to a smooth dentino-enamel junction that tends to be scalloped in DI patients [9]. Teeth affected by DI do not seem to be more susceptible to caries than normal teeth; in fact, the structure of dentin in teeth affected by DI, namely, the absence of ordinary dentinal tubules, suggests greater caries resistance [20]. Although the permanent teeth of the case presented here confirm this with its absence of dentinal caries, secondary caries was examined under the SSCs. The main reason of this situation may be the coronal microleakage of the prefabricated crowns.

Treatment of patients with DI should focus on protecting teeth from further wear. A multidisciplinary treatment strategy is required to restore appropriate vertical dimension and good esthetics while providing adequate soft tissue support to maintain a normal facial profile. The treatment strategy for this case was to reconstruct all upper and lower teeth with a fixed partial denture in order to protect the remaining hard tissue and achieve sufficient vertical dimensions for function and esthetics. Metal-ceramic crowns were selected for posterior teeth for both stability and economic reasons. A Co-Cr metal framework was fabricated using the rapid manufacturing system of DMLS. Laser sintering forms part of a family of new manufacturing technologies known as rapid prototyping [21]. Laser sintering has applications in a wide range of areas including industrial engineering, the military and aerospace sectors, and medical products. It is gaining popularity in dentistry at last years. Also, DMLS is simpler, more precise, and healthier than conventional manufacturing and can be remarkably cost effective [21]. Moreover, DMLS technology affords highly accurate production of fixed partial dentures with ideal marginal fit and excellent mechanical properties [13, 16]. While both Co-Cr and Ti fine powder alloy mixtures can be used to produce metal frameworks for use in dentistry, because of its ease of fabrication, Co-Cr is most commonly used over porcelain restorations. For this reason, Co-Cr metal-ceramic fixed partial dentures that were manufactured by DMLS technique were chosen for the rehabilitation of posterior region of the presented case.

The anterior teeth of the patient were restored with all-ceramic crowns for esthetic reasons. Zirconium porcelain was selected for its superior resistance when compared to other all-ceramic materials. The main reasons for the selection of zirconium porcelain in this case were to protect the decreased resistance of anterior teeth and to adapt the patient esthetically to the rearranged anterior guidance due to the increased vertical height.

## 4. Conclusion

Complicated cases such as DI require multidisciplinary treatment to achieve the best results. Early diagnosis and treatment of DI patients may preserve dental tissue and the stomatognathic system. Appropriate treatment may be required to prevent subsequent restorative problems. In the case presented here, laser sintering technology and a CNC unit were used together to achieve satisfactory function and esthetics, and the patient was recalled for periodical control to extend the longevity of his restorations.

## Figures and Tables

**Table 1:** Classification of osteogenesis imperfecta.

Type	DI	Clinical severity	Typical features
I	−	Mild, nondeforming OI	Normal height or mild short stature; blue sclera
II	?	Perinatal lethal	Multiple rib and long bone fractures at birth; pronounced deformities; broad long bones; low density of skull bones on radiographs; and dark sclera
III	+	Severely deforming	Very short; triangular face; severe scoliosis; and grayish sclera
IV	+	Moderately deforming	Moderately short; mild-to-moderate scoliosis; and grayish or white sclera
V	−	Moderately deforming	Mild-to-moderate short stature; dislocation of radial head; mineralized interosseous membrane; hyperplastic callus; and white sclera
VI	−	Moderate-to-severe deforming	Moderately short; scoliosis; accumulation of osteoid in bone tissue, fish-scale pattern of bone lamellation; and white sclera
VII	−	Moderately deforming	Mild short stature; short humeri and femora; coxavara; and white sclera

Modified from [5, 7]. DI: Dentinogenesis Imperfecta.
